# Draft Genome Sequence of *Kocuria* sp. Strain UCD-OTCP (Phylum *Actinobacteria*)

**DOI:** 10.1128/genomeA.00172-13

**Published:** 2013-05-09

**Authors:** David A. Coil, Jessica I. Doctor, Jenna M. Lang, Aaron E. Darling, Jonathan A. Eisen

**Affiliations:** University of California, Davis, Genome Center, Davis, California, USA

## Abstract

Here, we present the draft genome of *Kocuria* sp. strain UCD-OTCP, a member of the phylum *Actinobacteria*, isolated from a restaurant chair cushion. The assembly contains 3,791,485 bp (G+C content of 73%) and is contained in 68 scaffolds.

## GENOME ANNOUNCEMENT

Members of the *Kocuria* genus (formerly classified in the genus *Micrococcus*) have been isolated from numerous environments, including fermented food (1), plants (2), desert soil (3), marine sediments (4), and Antarctic cyanobacterial mats (5). *Kocuria* spp. are characterized as Gram positive, coccoid, and aerobic. While they are not normally considered pathogenic, some species have been implicated in bacteremia in immunocompromised patients (6).

*Kocuria* sp. strain UCD-OTCP was isolated from a restaurant chair cushion in Davis, CA, as part of an undergraduate project to provide microbial reference genomes from the built environment. The surface was wiped with a sterile swab, which was immediately streaked onto a Luria broth (LB) agar plate. After incubation at 37°C, single colonies were picked for serial dilution streaking and the organism was identified by Sanger sequencing of the 16S rRNA gene PCR product produced by the 1391R and 27F primers. Genomic DNA was extracted using a Wizard genomic DNA purification kit (Promega) from fresh overnight cultures. Illumina paired-end libraries were then made from sonicated DNA using a TruSeq DNA sample prep v2 kit (Illumina). Fragments between 300 and 600 bp were selected using a Pippin Prep DNA size selection system (Sage Science). A total of 4,074,016 paired-end reads were generated on an Illumina MiSeq, at a read length of 160 bp. Quality trimming and error correction of the reads resulted in 3,825,344 high-quality reads. All sequence processing and assembly were performed using the A5 assembly pipeline (7). This pipeline automates the processes of data cleaning, error correction, contig assembly, scaffolding, and quality control. The assembly produced 68 scaffolds (minimum, 488 bp; maximum, 254,872 bp; *N*_50_, 101,818 bp). During scaffolding, some contigs were merged based on short overlaps and read-pair information, yielding a final collection of 71 contigs that were submitted to GenBank. This resulted in a final assembly of 3,791,485 bp, with a G+C content of 73%, and an overall coverage estimate of 160×. Completeness of the genome was assessed using the PhyloSift software (A. Darling, G. Jospin, E. Lowe, E. Matsen, H. Bik, and J. Eisen, unpublished data), which searches for a list of 40 highly conserved single-copy marker genes (D. Wu, G. Jospin, and J. Eisen, unpublished data), all of which were found in this assembly.

Automated annotation was performed using the RAST annotation server (8). *Kocuria* sp. strain UCD-OTCP contains 3,432 predicted protein-coding sequences and 51 predicted noncoding RNAs. A phylogenetic tree of 160 cultured isolates of *Kocuria* spp. was produced using the Ribosomal Database Project (RDP) Web-based tool, which implements a weighted neighbor-joining tree-building algorithm (9). *Kocuria* sp. strain UCD-OTCP falls within a poorly resolved paraphyletic clade containing *Kocuria rosea* and *Kocuria polaris* (http://dx.doi.org/10.6084/m9.figshare.646175). Because the 16S rRNA gene sequence of *Kocuria* sp. strain UCD-OTCP has >99% identity to those of both of these other *Kocuria* species, and the phylogenetic relationships within the genus are unclear, we were unable to assign a species name to this isolate.

The genome sequences of only two other *Kocuria* species have been published, *Kocuria atrinae* (1) and *Kocuria rhizophila* (4). The 16S rRNA gene from *Kocuria* sp. strain UCD-OTCP has 97% identity to that from *K. atrinae* and 97% identity to that from *K. rhizophila*.

### Nucleotide sequence accession numbers.

This Whole-Genome Shotgun project has been deposited at DDBJ/EMBL/GenBank under the accession no. AOSQ00000000. The version described in this paper is the first version, accession no. AOSQ01000000. Illumina reads are available at http://dx.doi.org/10.6084/m9.figshare.157191.
